# Fabrication of SnO_2_-Reduced Graphite Oxide Monolayer-Ordered Porous Film Gas Sensor with Tunable Sensitivity through Ultra-Violet Light Irradiation

**DOI:** 10.1038/srep08939

**Published:** 2015-03-11

**Authors:** Shipu Xu, Fengqiang Sun, Shumin Yang, Zizhao Pan, Jinfeng Long, Fenglong Gu

**Affiliations:** 1 School of Chemistry and Environment, South China Normal University, Guangzhou 510006, P. R. China; 2 Key Laboratory of Theoretical Chemistry of Environment, Ministry of Education, South China Normal University, P. R. China; 3 Guangzhou Key Laboratory of Materials for Energy Conversion and Storage, P. R. China

## Abstract

A new graphene-based composite structure, monolayer-ordered macroporous film composed of a layer of orderly arranged macropores, was reported. As an example, SnO_2_-reduced graphite oxide monolayer-ordered macroporous film was fabricated on a ceramic tube substrate under the irradiation of ultra-violet light (UV), by taking the latex microsphere two-dimensional colloid crystal as a template. Graphite oxide sheets dispersed in SnSO_4_ aqueous solution exhibited excellent affinity with template microspheres and were in situ incorporated into the pore walls during UV-induced growth of SnO_2_. The growing and the as-formed SnO_2_, just like other photocatalytic semiconductor, could be excited to produce electrons and holes under UV irradiation. Electrons reduced GO and holes adsorbed corresponding negative ions, which changed the properties of the composite film. This film was directly used as gas-sensor and was able to display high sensitivity in detecting ethanol gas. More interestingly, on the basis of SnO_2_-induced photochemical behaviours, this sensor demonstrated tunable sensitivity when UV irradiation time was controlled during the fabrication process and post in water, respectively. This study provides efficient ways of conducting the in situ fabrication of a semiconductor-reduced graphite oxide film device with uniform surface structure and controllable properties.

Graphene serial carbon materials, including the graphite oxide (GO) and the reduced graphite oxide (rGO), have gained more and more attentions in recent years in the fields of battery^1^,^2^, supercapacitor^3^, photocatalysis^4^, gas sensor^5^,^6^, etc. Multi- or mono-layered GO sheets obtained by the chemical exfoliation of graphite possess many reactive oxygen-containing groups that can be further functionalized and controlled^7^,^8^,^9^, which expand the application of graphene serial materials significantly^10^,^11^. They are easy to incorporate with various types of functional materials, such as polymers^12^,^13^, biomaterials^14^, and many inorganic materials^15^,^16^,^17^. To actually enhance the performance of the composites, GO is usually required to be transferred into rGO with different reduced degrees (i.e. the graphitization degree) by a specific reduction process. rGO has partial characters of GO and graphene, and particularly, its property can be easily controlled to meet different applications. Therefore, taking the GO sheets as the initial component to obtain functional material-rGO composites is essential and promising in practical applications.

Using graphene serial materials into gas-sensor is a hot topic. In addition to pure graphene^18^,^19^, molecular-modified graphene^20^,^21^ and rGO gas-sensors^22^,^23^, many excellent semiconductor-rGO composite resistance-type gas sensors have been fabricated to detect various environmental gases^24^,^25^,^26^. Due to the incorporation of rGO sheets, heterojuctions can form, resulting in the enhanced sensing performances of the corresponding semiconductors, including the improved sensitivity and the decreased working temperature. By tuning the component ratio or reduced degree of GO or rGO during the fabrication process^5^, the sensitivity of the sensor can be controlled to a certain extent. However, just like the construction of many graphene serial composite devices, to fabricate the semiconductor-rGO sensor, the composite powders are usually fabricated firstly, processed into paste and then coated onto specific substrates. Besides the arduous procedure, this process inevitably causes the overlap of sheet-like composite slurries, loose contact among diverse composite sheets, and non-uniform surface that consequently affects the repeatability, the stability and the performance controllability of the devices. Moreover, once these sensors are constructed, no method could be employed to further tune the sensitivity, which restricts the optimization of a practical sensor. A method that directly incorporating the rGO sheets into a composite film and simultaneously patterning a uniform micro/nano-structured surface during film growth is thereby necessary but still a challenge.

Herein, we utilized a colloidal monolayer composed of closely packed polystyrene (PS) microspheres as the template to fabricate SnO_2_-rGO monolayer-ordered porous film gas sensor in aqueous solution under UV irradiation. GO sheets were hydrophilic and flexible in nature and exhibited excellent affinity with PS. They were easy to be incorporated and stabilized into the film during the formation of SnO_2_ around the PS microspheres. The resulting composite film was uniformly structured with the ordered arranged pores after removal of the template. More importantly and interestingly, the SnO_2_, similar to many photocatalytic semiconductors^27^,^28^, could produce photogenerated electrons and holes that modify the properties and surface states of GO, rGO or SnO_2_ itself under UV irradiation. Based on the photochemical behaviours, the gas sensitivity was thereby tuned during the fabrication process and post in water, which represents a new method in controlling the performance of gas-sensor. The commercially available gas-sensing ceramic tube with a curved surface and SnO_2_ (i. e. the most widely used gas-sensing material) was chosen as substrate and model material, respectively, demonstrating the universality of the fabrication method and the practicality of the new porous film.

## Results

### Character of the GO sheets used in this study

**Figure 1:**
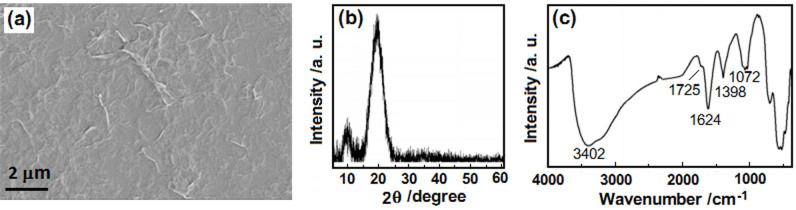
Characters of the as-prepared GO sheets used in this study. (a) SEM image of the sheets coated on a glass substrate; (b) XRD pattern; (c) FT-IR spectrum.

The GO sheets were specifically prepared by a simplified Hummer's method^29^. In the process, the oxidation time of the graphite was decreased and the ultrasonic processing was omitted, which was different from the general preparation of mono-layered GO sheets. As shown in Figure 1 (Fig. 1), the as-prepared GO sheets look like wrinkled and their edges are difficult to discern because of the very small size in thickness (Fig. 1a). The XRD spectra (Fig. 1b) exhibits two diffraction peaks appeared at 19.57° and 9.95°. This explained the GO sample prepared here principally contained two kinds of sheets with interlay space (d-spacing) of 0.45 and 0.88 nm. Relative to that of pure graphite, the peaks significantly shift towards lower angles, explaining GO sheets are considerably further apart from one another than in crystalline graphite (d_002 _ = 0.34 nm for graphite). The FT-IR spectrum (Fig. 1c) of GO shows the vibration band of the -OH groups at 3402 cm^−1^, O-H deformation peak at 1398 cm^−1^, a weak band at 1726 cm^−1^ assigned to C = O in carboxylic acid moieties and 1072 cm^−1^ due to the C-O-C stretching vibrations in epoxy and alkoxy groups^30^. These demonstrated that the surface of GO had been functionalized with different kinds of oxygen functional groups. The peak at 1624 cm^−1^ was assigned to the contributions from the skeletal vibrations of the graphitic domains, showing the remaining sp^2^ character^31^.

### Fabrication process of SnO_2_-rGO porous film

**Figure 2:**
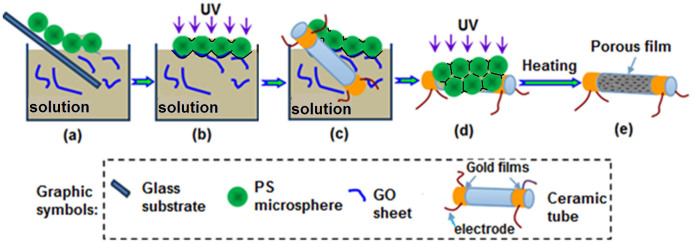
Schematic showing the fabrication of SnO_2_-rGO monolayer-ordered porous film gas sensor. (a) Transferring the colloidal monolayer from the glass substrate to the precursor solution; (b) irradiating the colloidal monolayer on solution with UV; (c) picking up the monolayer with a ceramic tube; (d) further irradiating the colloidal monolayer on the tube; (e) the final SnO_2_-rGO composite.

As shown in Fig. 2, by a transfer technique^32^, a colloidal monolayer of PS microspheres on the glass substrate was firstly transferred and floated on the surface of a homogeneous and transparent aqueous precursor solution containing GO sheets and SnSO_4_ (Fig. 2a). And then, UV irradiation was performed on the solution, which would induce photochemical reactions to generate GO-incorporated composite shells around the microspheres (Fig. 2b). Simultaneously, GO could be reduced to rGO. 30 min later, the produced shell-colloidal monolayer was picked up using a ceramic tube (Fig. 2c). It could wrap the tube because of the flexibility of the colloidal monolayer^33^,^34^. Voids in the film and in between the film and the substrate were still filled with solutions and rGO because of the capillary force. The ceramic tube was then placed under UV light for further irradiation (Fig. 2d). Photochemical reactions occurred again. Solid particles further formed and grew around the microspheres and on the tube surface, thus connecting the composite film with the tube. Finally, the ceramic tube was heated in a furnace for 1 h at 300°C. The PS microspheres were removed, leaving a stable SnO_2_-rGO monolayer-ordered porous film on the ceramic tube (Fig. 2e).

### Morphology and composition of the porous films

**Figure 3:**
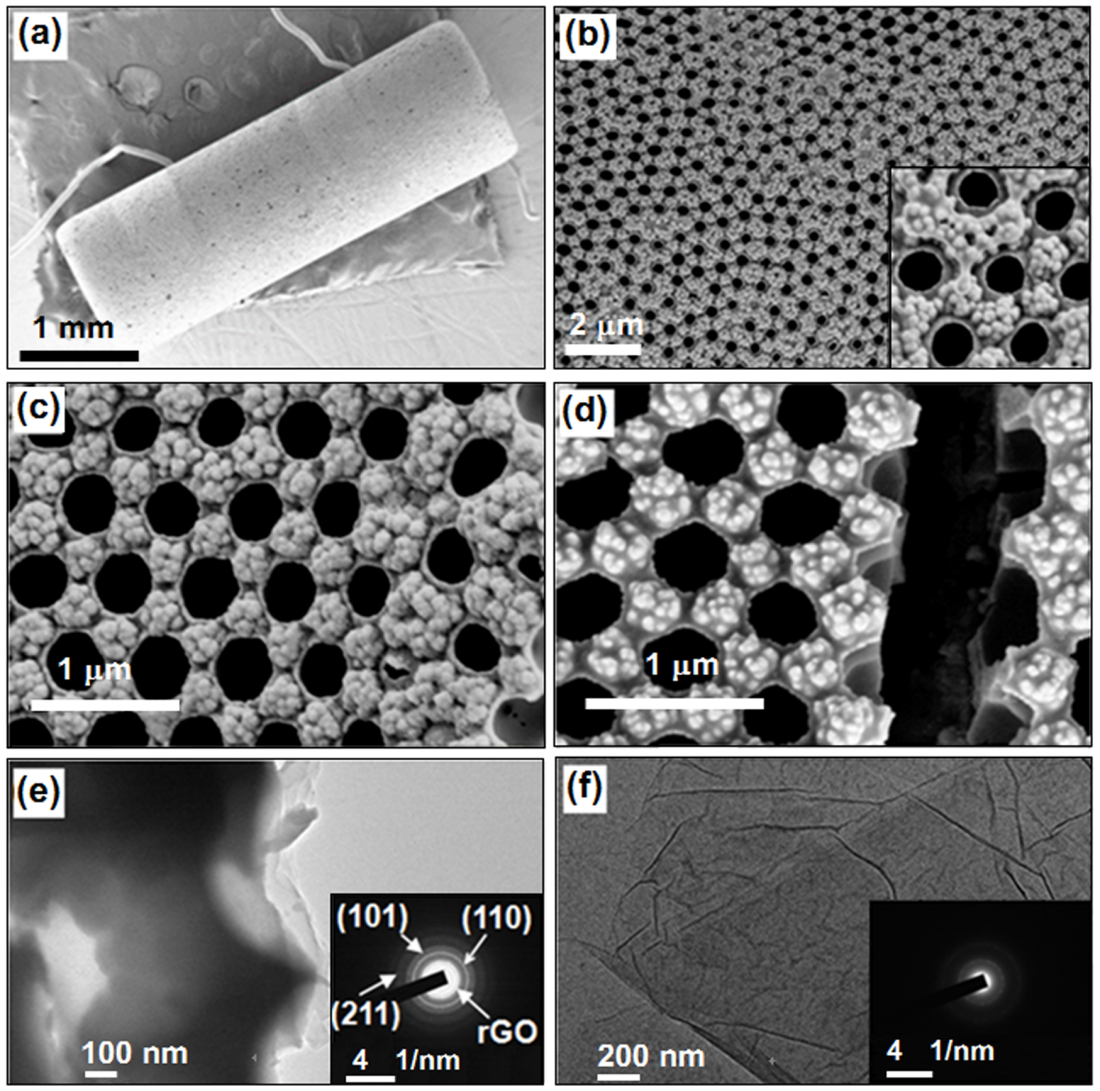
Morphologies of composite films and GO sheets. (a) A low magnification SEM image of SnO_2_-rGO film (Film-1.0) on the whole tube; (b)–(d) SEM images of Film-1.0, Film-0.5, and Film-4.0, respectively; (e) TEM image of Film-1.0; (e) TEM image of pure GO sheets.

Fig. 3 shows the morphologies of the SnO_2_-rGO monolayer-ordered porous films fabricated using a PS microsphere (750 nm in diameter) colloidal monolayer template. The total irradiation times, including the first (Fig. 2b) and the second (Fig. 2d) irradiation times, varied from 0.5 to 1.0, 2.0, and 4.0 h to obtain Film-0.5, Film-1.0, Film-2.0, and Film-4.0, respectively. Fig. 3a shows the SEM image of the ceramic tube covered with Film-1.0. The film covered the entire surface of the tube and connected the electrodes on the two ends. The pores in the film were closely arranged in a hexagonal pattern over a large area (Fig. 3b). The pores had circular openings with a diameter of ~ 350 nm and pore walls with a thickness of ~ 20 nm. The center-to-center spacing between the two adjacent pores was maintained at 750 nm, which is equal to the diameter of the template microspheres. Interestingly, some spherical particles with a size of ~ 85 nm were observed to have piled among three adjacent pores (inset of Fig. 3b). The principal morphologies cannot be modified by irradiation time, as shown in Film-0.5 (Fig. 3c) and Film-4.0 (Fig. 3d). Film-4.0 was randomly gashed several times with a sharp blade to investigate the inner morphology of the film further. An empty spherical pore chamber and a demarcation between the porous and particle layers were observed in a certain section (Fig. 3d). TEM image of Film-1.0 scraped off from the ceramic tube was shown in Fig. 3e. On the broken edge, sheet-like composites were found and they should form by the induction of GO sheets. The selected area electron diffraction (SAED) pattern displayed the polycrystalline structure of the composite. The (110), (101) and (211) planes of SnO_2_ were clearly discerned by the diffraction rings. Contrasting with the SAED pattern of pure GO sheets (inset of Fig. 3f), rGO in the composite could also be discerned. These explained the rGO sheets had been incorporated into the pore walls of the film.

**Figure 4:**
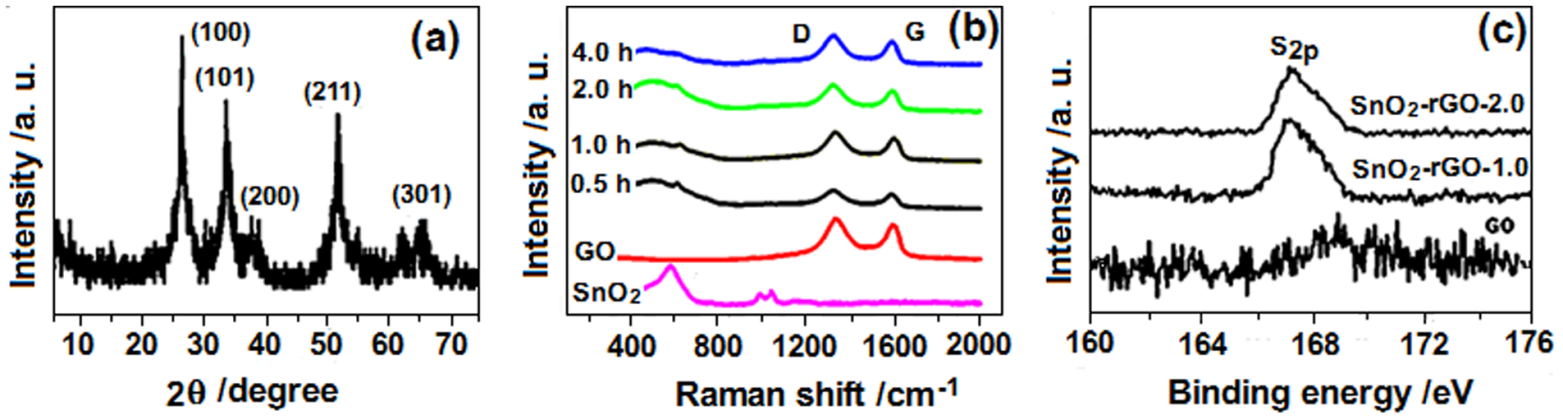
(a) XRD, (b) Raman, and (c) S_2p_ XPS spectra of the GO sheets and SnO_2_-rGO composites. For easy characterization, the corresponding porous films were prepared on the glass substrate under the same conditions as those on the ceramic tube and then they were scraped off to form into powders and measured.

Fig. 4a shows the X-ray diffraction (XRD) pattern of Film-1.0. All peaks exhibiting tetragonal-cassiterite structure belonged to SnO_2_ (JCPDS No. 21–1250). Other samples exhibited similar XRD patterns. The GO and rGO were discerned on the basis of the Raman spectra of the composites (Fig. 4b). Except for the pure SnO_2_ sample, the pure GO and SnO_2_-rGO composites (curves 0.5 h to 4.0 h) contained both G and D bands, showing that GO was introduced into the composites during fabrication. The intensity ratio of the D band to the G band (I_D_/I_G_) was calculated as 1.158 for GO. The obtained intensity ratios for the composites gradually increased from 1.176 to 1.191, 1.202, and 1.210 at irradiation times of 0.5, 1.0, 2.0, and 4.0 h, respectively. An increasing trend of the values was observed. The gradual increase in I_D_/I_G_ reflected the reduction degree of GO in the composites increased with the increase of UV irradiation time^35^,^36^. In addition, the S_2p_ XPS showed that the composite contained elemental sulfur that came from the adsorbed SO_4_^2−^ ions (Fig. 4c)^37^. An increase in irradiation time increased the sulfur content. For instance, when irradiation time was increased from 1.0 h to 2.0 h, the sulfur content increased from 2.46 to 3.02 At. %.

## Discussion

### Formation mechanism of the porous film

**Figure 5:**
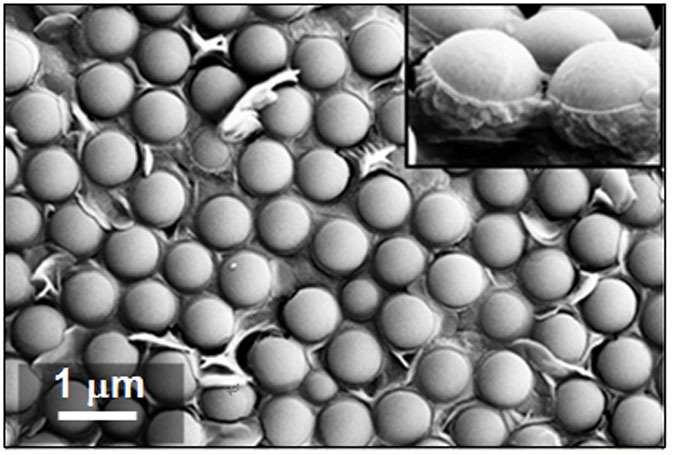
SEM image of a colloidal monolayer wrapped with GO sheets. The inset shows a lateral view of the monolayer.

GO sheets used here showed excellent hydrophilicity, affinity with PS microspheres and flexibility. To prove it, a colloidal monolayer template was floated onto the surface of a pure GO suspension (4.0 × 10^−5^ g/mL) by the same manipulation as that shown in Fig. 2a, and then it was directly picked up with a ceramic tube and dried at 60°C. As shown in Fig. 5, the interstices among PS microspheres had been filled with GO sheets. The lower part of PS microspheres immersed into the water had adsorbed some GO sheets due to the π-π stacking action^38^ and been wrapped (inset of Fig. 5). These characters provided the chance for the formation of rGO-incorporated porous films in aqueous solutions.

**Figure 6:**
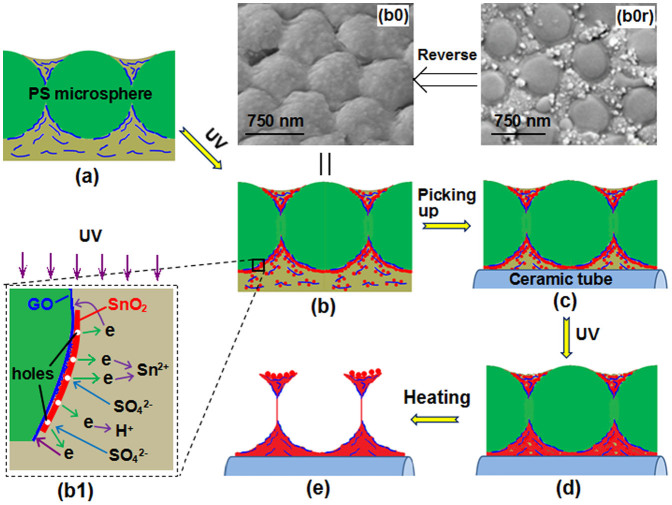
Formation process of SnO_2_-rGO monolayer porous film. (a) The existence modes of GO sheets; (b) SnO_2_/H_2_SnO_3_ particles was forming on the surface of PS microspheres and GO sheets under UV; (c) state of the colloidal monolayer and composite shells on the ceramic tube; (d) condensed composite film on the tube after the second irradiation; (e) SnO_2_-rGO monolayer porous film after removal of the PS microspheres; (b0) and (b0r) SEM image of the back side and the right side, respectively, of the composite film after irradiated 30 min; (b1) Photochemical behaviours induced by SnO_2_ under UV.

Obviously, when the colloidal monolayer was floated on the SnSO_4_ solution dispersed with GO sheets, the GO sheets would exist in three typical modes, i. e. wrapping the surface of microspheres, suspending near the microspheres and suspending above the colloidal monolayer (Fig. 6a). At the same time, GO sheets could adsorb Sn^2+^ ions by the charge attractions. During the UV irradiation (Fig. 6b), Sn^2+^ in the solution would adsorb photons to produce metallic Sn and Sn^4+^ (Eq. 1)^39^,^40^,^41^. The newly generated metallic Sn was then oxidized into SnO_2_ by oxygen in the air (Eq. 2); Sn^4+^ was hydrolyzed into H_2_SnO_3_ particles (Eq. 3).
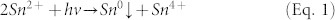

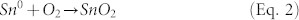

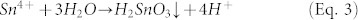


According to conventional nucleation theory, SnO_2_/H_2_SnO_3_ nuclei should preferably form and grow on the surfaces of GO and PS microspheres. Around the microspheres, shells could be established. The GO sheets that wrapped the PS microspheres were directly buried and naturally became compositions of the shells. An increase in UV-irradiation time caused the SnO_2_/H_2_SnO_3_ particles on the GO sheets near the PS microspheres to enlarge and bond with the particles on such microspheres. These GO sheets were also incorporated into the shells. For GO sheets above the colloidal monolayer, they were only minimally restricted of the voids among the microspheres. At least, one of the surfaces of a GO sheet was toward to the air. Functional oxygen-containing groups easily induced the nucleation and free growth of the SnO_2_/H_2_SnO_3_ particles on these surfaces. Such particle-GO sheets might be deposited onto the template or still floated on the solution surface. To confirm the formation process, the shell-colloidal monolayer composite film was watched after irradiated 30 min. Fig. 6b0 showed the morphology of the back side (towards to the solution) of the film, disclosing the spherical shells had formed. The right side (towards to the air) (Fig. 6b0r) showed the shells formed only around the lower part of the template microspheres immersed in the solution. The particles on surfaces were obviously bigger than those combined in the shells, which should be attributed to their comparatively free growths induced by the GO sheets existing above the template. The XRD pattern (not shown here) of such film was similar to that of the finally obtained film, which explained SnO_2_ could form under the UV irradiation.

After the composite film was picked up with the ceramic tube, the fundamental structure was not changed (Fig. 6c). Solution in the voids would be brought out. Once UV irradiated the film again, new SnO_2_/H_2_SnO_3_ nuclei would form and grow up in all voids, adhering to any surface to remedy the defects and connect the composite film with the ceramic tube (Fig. 6d). Simultaneously, water was converted to vapour, and the solution gradually gathered among the microspheres, carrying the particle-GO sheets suspending above the colloidal monolayer. During the final heating process, all tin ions and H_2_SnO_3_ were transferred into SnO_2_, particles on the surface of the film continuously grew up, the film were densified, the connection between the film and the ceramic tube was further enhanced, accompanied by the removal of PS microspheres (Fig. 6e). Monolayer porous film with piled particles among three adjacent pores was hence obtained (Fig. 3). Due to the high mechanical performance of GO, cracks in the film could be effectively avoided.

UV irradiation that was performed during the growth of SnO_2_ simultaneously induced the reduction of GO sheets and modified the surface state of SnO_2_. The as-formed SnO_2_ on the GO sheets, which served as excellent photocatalysts with a band gap of 3.2 eV, exhibited photocatalytic action and were excited by UV with a wavelength of ~ 254 nm to generate electrons (Eq. 4), leaving holes (h^+^) on the surface of the SnO_2_ particles (Fig. 6b1). These photogenerated electrons reduced not only Sn^2+^ to promote SnO_2_ formation, but also GO in situ in the composite (Eq. 5)^42^. The holes adsorbed the negatively charged SO_4_^2−^ ions in the solution. Increasing UV irradiation time caused more oxygen-containing groups on the GO to be removed and more SO_4_^2−^ ions to be adsorbed on the SnO_2_ surface.


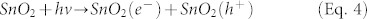

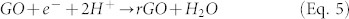


### Gas-sensitivity of the porous films

**Figure 7:**
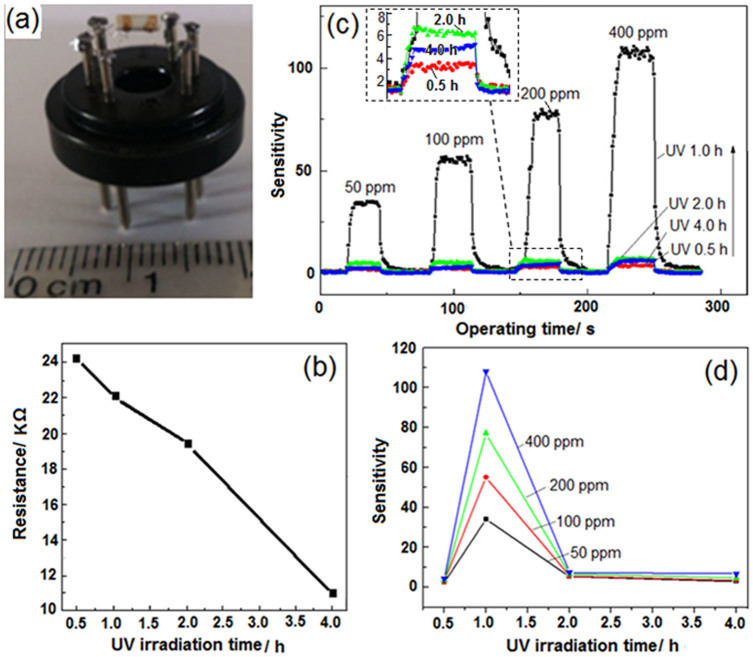
Different characterizations performed on the measurements of the film gas sensor. (a) A photo of the sensor to be measured; (b) variation of the resistance in air with UV-irradiation time; (c) variation of the sensitivity with measurement time involved in detecting the different concentrations of ethanol gas and (d) variation of the final sensitivity with UV-irradiation time. Response times of Film-1.0 are 6, 6, 7 and 8s in detecting 50, 100, 200 and 400 ppm ethanol gas, respectively. The recovery times are 8, 8, 6 and 6s, respectively. For other film sensors, the response time and the recovery time are all in the range of 3-8s.

The as-fabricated SnO_2_-rGO composite monolayer-ordered porous films on the ceramic tube were directly used as gas sensors to detect ethanol gas in air, after the electrodes and heating wire on the ceramic tube were welded on a specific support (Fig. 7a). The working temperature of the sensors was optimized at 175°C. At this temperature, the resistance of the sensors in air gradually decreased from 24.2 KΩ to 11.0 KΩ with increasing UV irradiation time from 0.5 h to 4.0 h during porous film formation (Fig. 7b). Gas sensitivity is defined as S = R_air_/R_gas_, where R_air_ and R_gas_ are the resistances of the sensor in the air and the air mixed with ethanol gas, respectively^43^. All sensors could quickly respond to the introduced ethanol gas, and the response time was always limited within 8 s in detecting different concentrations of gas (Fig. 7c). They also quickly recovered within 8 s after the gas was removed. For a specific sensor, sensitivity naturally increased with an increase in ethanol gas concentration. For instance, the sensitivities obtained for Film-1.0 sensor were 33.6, 55.6, 77.3, and 108.0 in detecting 50, 100, 200, and 400 ppm of ethanol gas, respectively. Different sensors exhibited varying sensitivity depending on the UV-irradiation time employed during fabrication. As shown in the inset of Fig. 7c and Fig. 7d, during the detection of any concentration of ethanol gas, the sensitivity of the sensors initially increased with an increase in irradiation time (from 0.5 h to 1.0 h) and then decreased with the continuous increase in irradiation time (from 1.0 h to 2.0 h and then 4.0 h). For instance, the detection of 200 ppm ethanol gas yielded the following sensitivities: 3.5, 77.3, 6.6, and 4.8, which were obtained from Films-0.5, -1.0, -2.0, and -4.0, respectively. The sensitivity of the porous films was obviously controlled by UV-irradiation time during fabrication.

**Figure 8:**
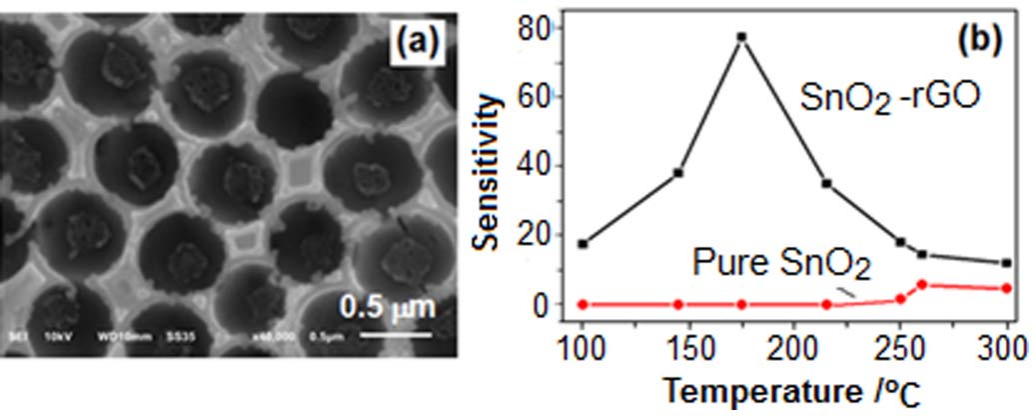
(a) SEM image of pure SnO_2_ monolayer porous film; (b) Sensitivities of SnO_2_-rGO and pure SnO_2_ monolayer porous film sensor varied with the working temperature in detecting 200 ppm ethanol gas. The two films were obtained by the same procedure.

Additionally, compared with the pure SnO_2_ porous film sensor, the composite sensor also had highly increased sensitivity and decreased working temperature because of the introduction of rGO. Figure 8a showed the morphology of a pure SnO_2_ sensor fabricated under the same conditions as that of Film-1.0. It had the similar porous structure to SnO_2_-rGO monolayer porous film. When it was used to detect 200 ppm ethanol gas, no signal could be obtained below 250°C (Fig. 8b). At 260°C, it showed the maximum sensitivity of 3.9. However, for the SnO_2_-rGO sensor, it could response to ethanol gas at ~ 100°C; its sensitivity had already reached the maximum value of 77.3 at 175°C; the sensitivity had increased nearly 20 times, while the working temperature was decreased 85°C. There were obvious advantages for the SnO_2_-rGO sensor in practical application.

### Reasons for the variation of the sensitivity

Generally, a SnO_2_-based sensor that is exposed to air will cause O_2_ molecules to be chemisorbed. These O_2_ molecules then capture some electrons of SnO_2_ and are converted to O_2_^−^, O^−^, and O^2−^ on the sensing body surfaces^44^. After a reducing gas (e.g., ethanol gas) is introduced, some oxygen species will be reduced and removed from the surfaces above a certain temperature, resulting in the release of the captured electrons, decrease in the resistance of SnO_2_, and display of sensitivity (R_air_/R_gas_). Therefore, the quantity of adsorbed oxygen species causes variations in the sensitivity. In the composite system, SnO_2_ particles and rGO nanosheets formed hetero-junctions. During continuous UV-irradiation, the GO continuously accepted electrons in the solution, while the SnO_2_ generated holes (Eq. 1) because of its photocatalytic action. This raised the chance of the rGO electrons to diffuse through the heterojunction into the SnO_2_ layer. The exposure of such final composite film to air enabled oxygen to extract more electrons easily, and the quantity of adsorbed oxygen species was thereby increased. However, some photogenerated holes on the surface of the SnO_2_ adsorbed the SO_4_^2−^ ions (Fig. 4c and Fig. 6b1). The adsorbed SO_4_^2−^ ions bear an electron-rich group that hindered the diffusion of electrons from rGO, repelled oxygen, and occupied some sites on the surface of the final film, thereby reducing the chance for adsorbing oxygen species. These two factors, which are mutually contradictory, are responsible for the variations of the sensitivity of the final sensors. When UV-irradiation time in the fabrication process was increased from 0.5 h to 1.0 h, the number of extractable electrons in the rGO constantly increased, while the number of adsorbed SO_4_^2−^ ions on the surface of SnO_2_ remained limited. The electron-induced adsorption for oxygen species in the final composite film prevailed, thus resulting in increased sensitivity with the increased UV-irradiation time. After 1 h, an increase in the quantity of SO_4_^2−^ ions adsorbed by the holes was observed (Fig. 4c). Such increase led to the decreased sensitivity.

### Post tuning the sensitivity of the sensor

**Figure 9:**
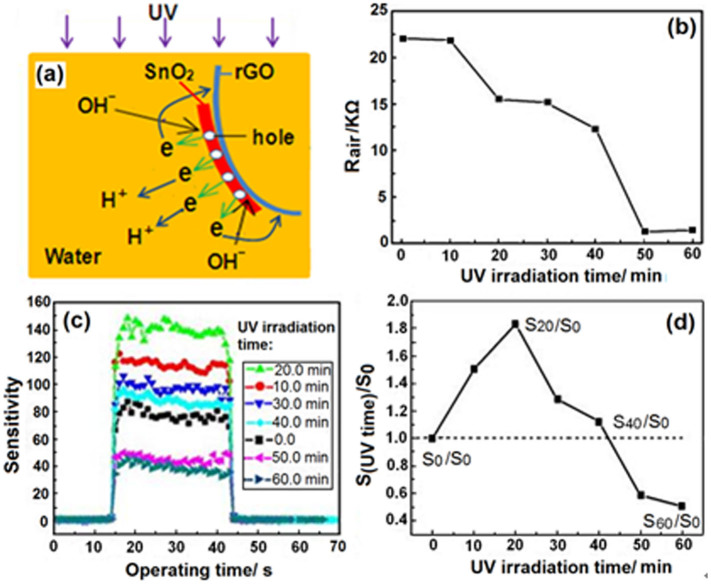
(a) Photochemical behaviors that occurred when the as-fabricated sensor was post irradiated with UV light in water; (b) the resistance variation of the sensor from Film-1.0 in air with the UV irradiation; (c) responses of the sensor after irradiated different times to 200 ppm ethanol gas; (d) the ratios between the sensitivities of sensor after and before different UV-irradiation times.

More interestingly, the sensitivity of the as-fabricated SnO_2_-rGO sensor could be post-tuned by additionally irradiating the sensor with UV light in deionized water. The sensor fabricated using Film-1.0 was used as an example to illustrate this process. Every after 10 min of UV irradiation, the sensor was taken out and dried, and its response to 200 ppm ethanol gas was measured. The film grown in the precursor solution contained many oxidizing ions (e. g., H^+^, Sn^2+^, and Sn^4+^), which was in contrast to the as-formed film in water that was surrounded with only few H^+^ and OH^−^ (Fig. 9a). SnO_2_ in the as-formed film, under UV light with the same light intensity, was also excited, and nearly all the photogenerated electrons were accepted by the rGO. The rGO was rapidly reduced further, and the accumulation rate of the electrons in the composite increased significantly, resulting in the rapid decrease in resistance of the sensor in air (Fig. 9b). Few electrons were accepted by water to produce H_2_ and OH^−^. The new photogenerated holes adsorbed the OH^−^ ions, but most of these holes induced the diffusion of more electrons from rGO to SnO_2_ at the start of the process (from 0 to 20 min). This process provided more extractable electrons in the sensing body. These electrons can adsorb more oxygen species onto the surface of the final sensor. Although the additionally adsorbed OH^−^ ions had negative effects, the total quantity of adsorbed oxygen increased, resulting in the gradual increase in sensor sensitivity. The sensitivity (S_20_) of the sensor exposed to UV light for 20 min was nearly twice that (S_0_) prior to irradiation (Fig. 9c and 9d). A decrease of the oxygen-containing groups in rGO after 20 min of irradiation caused more electrons to be accepted by water, producing more absorbable OH^−^ ions. Similar to the case of SO_4_^2−^, the adsorbed OH^−^ ions hindered the adsorption of O_2_. As a result, the sensitivity of the sensor gradually decreased with increasing UV irradiation time. The sensitivity decreased to approximately 0.5 times that of the original after 50 min and then remained nearly steady.

Obviously, this technique for post-tuning the sensitivity of the composite sensor in water based on the photocatalytic reduction of GO by semiconductor is very practical. Many semiconductors used in gas-sensor possess photocatalytic activities as well, such as Fe_2_O_3_, CdS, ZnS, ZnO, CuO, etc. Once they are combined with GO or rGO by various suitable methods to constitute corresponding sensors, the sensitivities could be systematically tuned by UV light irradiation. This will help to obtain the gas-sensor with more excellent performances.

## Conclusions

In conclusion, a template-assisted UV irradiation method had been exemplarily utilized for direct fabricating SnO_2_-rGO monolayer-ordered porous film gas sensors on a ceramic tube. GO sheets were incorporated in situ and gradually reduced during the formation of SnO_2_. Because of the SnO_2_-induced photochemical behaviours, the reduced degree of GO and the amount of negative ions adsorbed on the surface of SnO_2_ increased with the increase of UV irradiation time. The sensitivity of the final SnO_2_-rGO films to ethanol gas was thereby tuned by controlling the irradiation during the fabrication process and post-tuned in water. The proposed template method is novel, easily manipulated, low cost, and can be applied to the universal fabrication of various semiconductor-rGO composite films with uniform surface microstructures on various solid substrates. Controlling of the performance (induced by the resistance, heterojunction or surface state) of the composite film by utilizing the photocatalytic action of the semiconductor can efficiently and flexibly optimize the rGO-incorporated semiconductor device according to the practical applications.

## Methods

### Fabrication of GO sheets

The GO sheets were prepared by a simplified Hummers method. Briefly, 1.0 g powdered flake graphite and 0.6 g NaNO_3_ were added into 23 ml concentrated H_2_SO_4_ into a flask cooled in an ice-bath under agitation. And then 3 g KMnO_4_ was added to the suspension slowly maintaining the vigorous agitation. The flask was taken out and put into a water bath at a temperature of 35°C. 30 min later, 30 ml deionized water was slowly stirred into the paste to form a suspension. After 1.0 hr, the suspension was further diluted with 27 ml water and then 3 ml 30% H_2_O_2_ was dropped into to reduce the residual KMnO_4_ and MnO_2_. Subsequently, the suspension was centrifuged at 3000 rpm for 4 minutes. The upper yellow grease was extracted and washed with water. The GO sheets were finally obtained.

### Fabrication of monolayer colloid crystal

Monodispersed PS-microsphere (750 nm in diameter) suspensions (2.5 wt % in water, surfactant free) were bought from Alfa Aesar Company. The ordinary glass substrate (1.5 × 1.5 cm^2^) was ultrasonically cleaned in acetone and then in ethanol for 1 h. Subsequently, the substrate was mounted on a custom-built spin coater. An amount of 10 μL of PS-microsphere suspensions was dropped onto the substrate. Large area monolayer (more than 1 cm^2^) colloidal crystal could be fabricated by a spin-coating method at a speed of 800 rotations per minute.

### Fabrication of SnO_2_-rGO monolayer-ordered porous film

The precursor solution was prepared by dissolving 0.108 g SnSO_4_ in 12 mL 4.0 × 10^−5^ g/mL GO homogeneous aqueous dispersion. The template was floated on the surface of the precursor solution and irradiated with two UV lamps (8 W, 254 nm, 0.17 mW**·**cm^−2^) for 0.5 h. Subsequently, it was picked up with a commercially supplied ceramic tube (2 mm in outer diameter and 5 mm in length) to undergo another round of irradiation under the same UV lamps. Finally, the ceramic tube was dried and heated at 300°C in a furnace to remove the template.

### Gas-sensing test

The gas sensing test was performed on a WS-30A system (Weisheng Instruments Co., Zhengzhou, China). A stationary gas-distribution method was used for gas response testing at 175°C. The ethanol gas to be detected was injected into an inclosed test chamber and mixed with air. The conductivity (or resistance) of a sensor would be changed. After it was stabilized, the chamber was opened and the ethanol gas was removed. The conductivity would be recovered. The same procedure was followed for the recycling test.

### Characterizations

The morphologies of the resulting porous films on the ceramic tubes were examined by scanning electron microscopy (SEM, Shimadzu SS-550 and Quanta 250 FEG). The compositions were characterized by X-ray powder diffraction (XRD, D/max2200, with Cu-K a radiation), Raman Spectrometer (Nicolet NXR 9650) and X-ray photoelectron spectrometer (XPS, ESCALAB 250). Samples for the XRD, Raman and XPS measurements were prepared on the glass substrates under the same conditions as that prepared on the ceramic tubes.

## Author Contributions

S.X. and F.S. designed and performed all experiments and co-wrote the paper. S.Y. contributed to Fig. 1. Z.P. contributed to Fig. 7. J.L. contributed to the analysis of XPS. F.G. contributed to the correction and polish of the whole manuscript. All authors reviewed the manuscript.
